# DRABAL: novel method to mine large high-throughput screening assays using Bayesian active learning

**DOI:** 10.1186/s13321-016-0177-8

**Published:** 2016-11-10

**Authors:** Othman Soufan, Wail Ba-Alawi, Moataz Afeef, Magbubah Essack, Panos Kalnis, Vladimir B. Bajic

**Affiliations:** 1 Computational Bioscience Research Center (CBRC), King Abdullah University of Science and Technology (KAUST), Thuwal, 23955-6900 Saudi Arabia; 2 Infocloud Group, Computer, Electrical and Mathematical Sciences and Engineering Division (CEMSE), King Abdullah University of Science and Technology (KAUST), Thuwal, 23955-6900 Saudi Arabia

## Abstract

**Background:**

Mining high-throughput screening (HTS) assays is key for enhancing decisions in the area of drug repositioning and drug discovery. However, many challenges are encountered in the process of developing suitable and accurate methods for extracting useful information from these assays. Virtual screening and a wide variety of databases, methods and solutions proposed to-date, did not completely overcome these challenges. This study is based on a multi-label classification (MLC) technique for modeling correlations between several HTS assays, meaning that a single prediction represents a subset of assigned correlated labels instead of one label. Thus, the devised method provides an increased probability for more accurate predictions of compounds that were not tested in particular assays.

**Results:**

Here we present DRABAL, a novel MLC solution that incorporates structure learning of a Bayesian network as a step to model dependency between the HTS assays. In this study, DRABAL was used to process more than 1.4 million interactions of over 400,000 compounds and analyze the existing relationships between five large HTS assays from the PubChem BioAssay Database. Compared to different MLC methods, DRABAL significantly improves the F_1_Score by about 22%, on average. We further illustrated usefulness and utility of DRABAL through screening FDA approved drugs and reported ones that have a high probability to interact with several targets, thus enabling drug-multi-target repositioning. Specifically DRABAL suggests the Thiabendazole drug as a common activator of the NCP1 and Rab-9A proteins, both of which are designed to identify treatment modalities for the Niemann–Pick type C disease.

**Conclusion:**

We developed a novel MLC solution based on a Bayesian active learning framework to overcome the challenge of lacking fully labeled training data and exploit actual dependencies between the HTS assays. The solution is motivated by the need to model dependencies between existing experimental confirmatory HTS assays and improve prediction performance. We have pursued extensive experiments over several HTS assays and have shown the advantages of DRABAL. The datasets and programs can be downloaded from https://figshare.com/articles/DRABAL/3309562.

**Graphical abstract:**
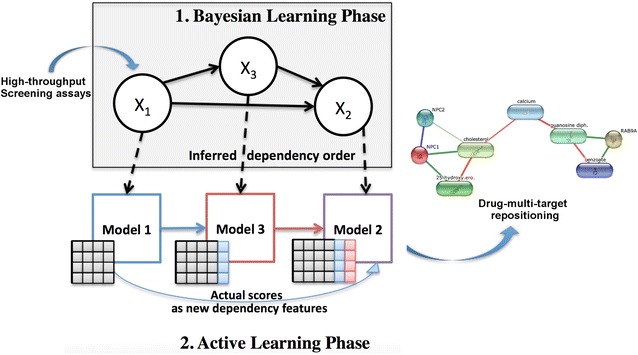
.

**Electronic supplementary material:**

The online version of this article (doi:10.1186/s13321-016-0177-8) contains supplementary material, which is available to authorized users.

## Background

 An unprecedented growth in biomedical data has surged in recent years. The ability to analyze big amounts of this data shall enable many opportunities that will, in turn, impact the future of healthcare [1]. It appears that, an era where personalized medicine, diagnostics and treatments are being adapted to everyday life, is on the horizon [2]. Yet, such growth opens challenges for developing data driven solutions that can effectively enhance decision-making in this foreseen healthcare environment.

Mining high-throughput screening (HTS) assays, for example, can provide highly valuable findings for novel uses of existing drugs or proposing new drugs with specific biological effects [3]. Revealing such previously unknown patterns may possibly significantly reduce costs [4] and speed up the drug development process. Yet many challenges, hinder the development of suitable methods for extracting useful information [5].

A wide variety of databases, methods and solutions were proposed towards handling the challenges that accompany the process of drug discovery by means of virtual screening. Virtual screening is a process based on using computational methods to identify chemical compounds that have high chance to interact with a specific biological target [6]. One common class of solutions to perform virtual screening is based on target prediction approaches that have been addressed by several studies [7–10]. Based on existing bioactivity information, target prediction helps in inferring novel molecular targets for known drugs [10]. Recently, 3D chemical similarity metrics and network algorithms were combined to achieve structure-based target prediction and reveal the binding mode of certain small molecules [11].

Several data mining models have been developed for chemical-target interactions [12–15]. These approaches differ from virtual screening, which rely on ligand–protein docking [16], as they do not require any prior knowledge of 3D structures of the target and its ligand. In addition, when these models are trained, they can be used for screening the biological activity status of a set of chemicals faster than ligand–protein docking approaches [17]. Also, several web tools have been developed [18–21] that predict chemical-protein interactions.

Towards handling larger HTS assays and exploiting the set of common active interactions as a factor for improving classification performance, we explore formulating the problem as a multi-label classification (MLC) instead of the conventional binary classification setup. In data mining, MLC is receiving a noticeable attention in recent years, since good impact has been achieved in several studies [22–24]. MLC classification as compared to binary classification or multi-class classification attempts to take advantage of any possible dependency between the target classes in order to improve the prediction accuracy [25, 26]. Recently, there have been a number of studies showing advantage of using MLC classification in several problems related to biology [27–29]. MLC classification was used for modeling cross-resistance information between a set of drugs in order to enhance the prediction of a particular drug resistance in the human immunodeficiency virus (HIV-1) [29]. In order to realize a better understanding of the function of chloroplast proteins, a proposed MLC algorithm was applied in prediction of protein subchloroplast locations in chloroplast organelle [27]. It was also shown that when the MLC approach is compared to a single label classification, it coherently reflects the actual metabolism information when applied over a collection of CYP450 substrates [30]. Multi-label Naïve Bayes classification models were constructed to improve target prediction for relevant target proteins over a wide set of chemical compounds [31]. Other works, as well, have shown successful usage of MLC to predict how molecules interact and analyze their biological activities [32, 33]. A popular solution for MLC classification problems is known as the binary relevance (BR), where a binary classifier is trained separately for each target class label. While BR fails to take advantage of any dependency between the labels in a dataset, it is known to be generally quite accurate [34, 35]. Another state-of-the-art extension for BR that takes into account the dependency between the labels are classifier chains (CC) [36]. The lack of completely labeled training instances, imposes substantial challenges for MLC classification, especially in that most of the proposed relevant solutions do not deal with this problem [37]. In our confirmatory HTS BioAssay datasets extracted from the PubChem BioAssay Database [38], we have positive and negative assigned interactions. Having both types of interactions is common in MLC problems. Yet, in our case, we have many missing interaction cases where the activity of a compound is not tested in a particular assay. Missing labels among the target classes (i.e. BioAssays) makes the MLC problem more challenging.

In this study, we developed DRABAL as a novel MLC solution based on Bayesian active learning. In DRABAL, we incorporate structure learning of a Bayesian network (BN) as a step to model dependency between the HTS assays. This structure can then be used to guide propagation of feedback between classifiers (also known as active learning), and to enhance prediction accuracy over individual binary classifiers. We used DRABAL to process more than 1.4 million interactions of 400,000 compounds and analyze the existing relationships between five large HTS assays from the PubChem BioAssay Database. We enabled drug-multi-target repositioning to show the utility of our method by screening against several targets all drugs from the DrugBank database [39] approved by U.S. Food and Drug Administration (FDA).

## Results and discussion

### Performance evaluation

F_1_Score is a performance evaluation measure. It computes the weighted average of sensitivity and precision [40]. It can be also referred to as balanced F-Score. In the context of HTS, a novel prediction relates to a suggested positive interaction whose confirmation requires experimental validation. In such a scenario precision is very important since a higher precision score reflects a lower number of false positives and thus, experimental validation costs are minimized. Therefore, we use F_0.5_Score as another summary measure that weighs precision twice as much as sensitivity [41, 42]. Finally, we use the geometric mean of sensitivity and specificity (GMean), to summarize prediction accuracy over both the true positive as well as the true negative rates.

Since a prediction in the case of MLC classification problems represents a subset of labels, different types of performance metrics are suggested [29]. Given indices of samples with actual positive assigned labels $A_{j}^{ + }$ for the $j$-th class label and corresponding set of indices with predicted positive labels $Y_{j}^{ + }$ for a total of $M$ samples, we define performance metrics using Eqs. (1)–(6). $A_{j}^{ - }$, and $Y_{j}^{ - }$ corresponding to the negative labels (i.e. negative interactions, see below) case and $A_{j}$ and $Y_{j}$ without superscripts denote indices of all relevant samples with positive and negative interactions, respectively. Negative labels mostly relate to inactive outcomes of the tested compounds in relation to the setup of a particular BioAssay, but since they may indicate an opposite phenotype of interaction (e.g. inhibition vs. activation) in the same assay, we call them negative interactions. These measures are based on computing the performance metric of interest for each target class label, and then averaging them for the $N$ class labels. This is a common performance evaluation approach for MLC classification problems [25, 29].

1 $$Sensitivity = \frac{1}{N}\mathop \sum \limits_{j = 1}^{N} \frac{{\left| {A_{j}^{ + } \cap Y_{j} } \right|}}{{\left| {A_{j}^{ + } } \right|}}$$

2 $$Specificity = \frac{1}{N}\mathop \sum \limits_{j = 1}^{N} \frac{{\left| {A_{j}^{ - } \cap Y_{j} } \right|}}{{\left| {A_{j}^{ - } } \right|}}$$

3 $$Precision = \frac{1}{N}\mathop \sum \limits_{j = 1}^{N} \frac{{\left| {A_{j} \cap Y_{j}^{ + } } \right|}}{{\left| {Y_{j}^{ + } } \right|}}$$

4 $$GMean = \sqrt {Sensitivity \times Specifcitiy}$$

5 $$F_{1} Score = \frac{{2 \times \left( {Precision \times Sensitivity} \right)}}{Precision + Sensitivity}$$

6 $$F_{0.5} Score = \frac{{1.25 \times \left( {Precision \times Sensitivity} \right)}}{0.25 \times Precision + Sensitivity}$$

Fivefold cross-validation is used in our computational experiments. Fivefold cross-validation is considered suitable for computing a non-biased score estimate [43] and we chose it due to the large number of interactions in our HTS assay datasets (as shown in Table 4). In order to test the significance of difference between the examined methods, we used the pair-wise *t* test at the 5% significance level.

### Performance comparison

Here, we describe results of our experimental studies over five large HTS assays composed of more than 1.4 million interactions and more than 400,000 chemical compounds from the PubChem BioAssay Database [38]. The experiments are designed to specifically test the advantage of employing dependencies between these assays for improved prediction accuracy. In order to achieve this, we have considered several comparisons. We compared our solution with BR, the most widely used for MLC classification [44]. BR is known also as a very hard baseline to beat, especially when the number of target labels is considerably small [36]. For BR, we have selected support vector machines (SVM), random forest (RF) and *K*-nearest neighbors (KNN) as base classifiers for training models for each label. We call these benchmark methods BR-SVM, BR-RF and BR-KNN, respectively. BR methods do not handle samples with missing labels. They just ignore any such case and exclude it from the data.

Another MLC solution that exploits dependencies between target classes for multi-label prediction is based on classifier chains (CC) [36]. In CC, once a classifier is built for one target label, this label is added to the feature and used for training of the next classifier in a chain order and so on. CC does not deal directly with missing labels that characterize the multi-label HTS assay datasets we have. In order to apply CC over the datasets, we assume all compounds that did not have any reported interaction for a specific assay, to have a negative label in the training set. Treating missing labels as negative labels is one of the approaches of handling missing labels in MLC classification [45]. It should be noted that this step is taken only for CC, but in our method we handle missing labels differently using active learning, which helps in quantifying a probability score of interaction for each missing case instead of assuming it to be negative. Using this approach, we extend CC to handle missing labels and call it ‘classifier chains with missing labels extension’ (CC-MLE). As a base classifier, we choose RF for CC-MLE and DRABAL since RF outperformed the classification performance of SVM and KNN classifiers.

Table 1 shows a summary of the fivefold cross-validation comparison results for the five HTS assays. Using a typical fivefold cross-validation, the HTS assays data is partitioned into five approximately equally sized mutually distinct subgroups such that a single subgroup representing 20% of the data is retained for testing only and is not used in any way for developing the model. For each partition (fold) of the data, the model is developed on the training portion and evaluated on the testing portion. The results from the testing folds are averaged to produce an estimation of performance. Based on all summary evaluation metrics, DRABAL significantly outperformed other state-of-the-art methods. DRABAL improved the F_1_Score by about 22% on average when compared to other methods. For the F_0.5_Score that gives more preference to precision, DRABAL achieved the highest score with an average improvement of 23%. This confirms that DRABAL maintains enhancing both sensitivity and precision. For GMean, DRABAL also achieves the highest performance. Similar improvements were achieved by DRABAL when tested on a larger number of datasets (see Additional file 1: Table S1 and Table S2). Additional file 2 provides extensive comparisons using other validation methods. Using holdout validation, when training splits ranging from 80% to only 20% of the original data are used, DRABAL achieved the highest results in all cases. On average, DRABAL improved F_1_Score in absolute measures by 6.8 and 22.24% when compared to BR-RF and CC-MLE, respectively. These result in the relative improvements of DRABAL’s F_1_Score over BR-RF and CC-MLE of 19.1 and 108.28%, respectively. Also, using plots of performance over distance, for each of different 20 distance ranges DRABAL attained the highest F_1_Score.

**Table 1:** Comparison of methods across five different datasets using the fivefold cross validation

Method	GMean (%)	F_1_Score (%)	F_0.5_Score (%)
BR-SVM	46.04	28.84	34.39
BR-KNN	24.59	14.91	23.26
BR-RF	55.56	45.35	61.26
CC-MLE	40.79	28.59	46.86
DRABAL	61.05^a^	51.11^a^	64.52^a^

The HTS assays data is partitioned into five approximately equally sized mutually distinct subgroups such that a single subgroup representing 20% of the data is retained for testing only. For each partition (fold) of the data, the model is developed on the training portion and evaluated on the testing portion. The results from the testing folds are averaged to produce an estimation of performance. Statistically significant difference when compared with all other methods over fivefolds using *t*-test at the 5% significance level is denoted by ^a^

In order to recognize the specific effect of exploiting dependency between the HTS assays over having a single binary classifier for each dataset, we consider more closely the comparison with BR-RF. It is worth mentioning that RF was the base classifier used for BR-RF and DRABAL with exactly the same parameters and initializations. Moreover, using a Bayesian network to define proper dependencies between the assays, DRABAL only expands the set of original features by two new features, on average for each dataset. Out of 1064 original features, this change is only equivalent to 0.1%. In other words, there is no extreme difference between the conditions of the input data as well as the parameters of RF classifier in BR-RF and DRABAL methods. Nevertheless, DRABAL significantly (based on t-test) outperformed BR-RF increasing performance in absolute measures by about 5.5, 6 and 3.3% for GMean, F_1_Score and F_0.5_Score, respectively. This makes relative improvement of DRABAL’s performance over BR-RF of 9.88, 12.7 and 5.3% for GMean, F_1_Score and F_0.5_Score, respectively. This clearly confirms the contribution of considering common active interactions between the HTS assays as a dependency factor towards enhancing classification performance.

**Fig. 1:**
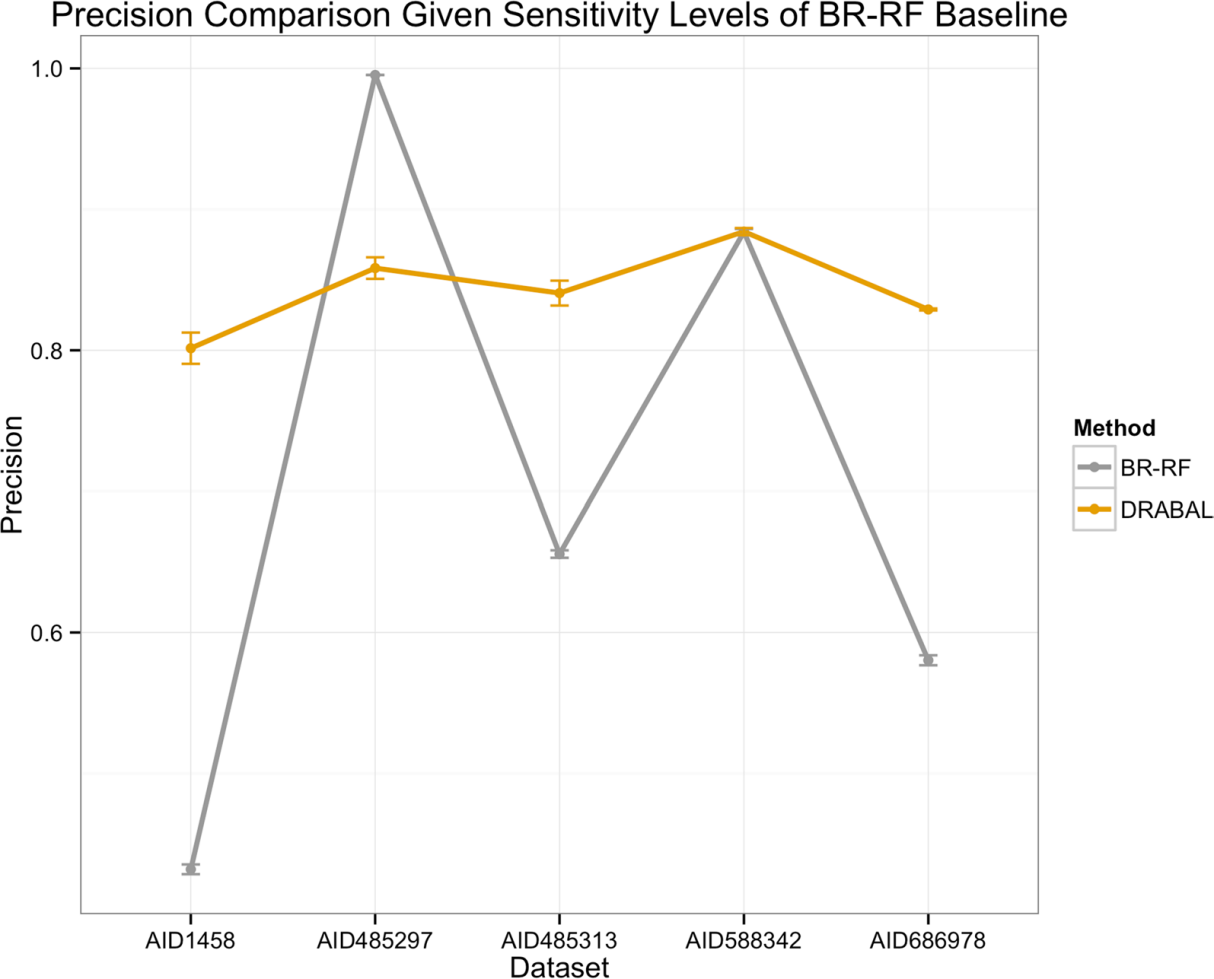
Precision comparison of DRABAL and BR-RF over five HTS assays. Precision is evaluated at the sensitivity levels of BR-RF (the second best method) in order to highlight achieved gain using DRABAL

Figure 1 illustrates the performance in terms of precision for every individual dataset when sensitivity is fixed at the same level the second best solution achieves (i.e. BR-RF). This indicates the gain we reach by reducing the number of false positives and thus, total experimental validation costs are minimized. As the orange color highlights, DRABAL improved precision largely in three out of five cases and achieves the same precision in one case.

Another experiment we perform is based on running different random initializations for ordering dependencies of targets in CC method and, follow the step proposed by DRABAL for handling missing labels. Thus, when running CC method, missing labels are not assumed negatives as in CC-MLE, but rather, a probability score of interaction is assigned the same way DRABAL does. This helps in measuring the advantage of, in particular, employing BNs into our algorithm. As Table 2 shows, based on *t*-test, DRABAL significantly outperformed average performance of ten random initialization of CC method. On average, DRABAL improved in absolute measures GMean, F_1_Score and F_0.5_Score by 9.91, 14.27 and 18.08%, respectively. This produces relative improvements of DRABAL’s performance (compared to the average performance of 10 random initialization of CC method) of 19.4, 38.7 and 38.9% for GMean, F_1_Score and F_0.5_Score, respectively.

**Table 2:** Comparison of methods across five different datasets using fivefold cross validation

Method	GMean (%)	F_1_Score (%)	F_0.5_Score (%)
RandomOrder-10	51.14	36.84	46.44
DRABAL	61.05^a^	51.11^a^	64.52^a^

The HTS assays data is partitioned into five approximately equally sized mutually distinct subgroups such that a single subgroup representing 20% of the data is retained for testing only. For each partition (fold) of the data, the model is developed on the training portion and evaluated on the testing portion. The results from the testing folds are averaged to produce an estimation of performance. Statistically significant difference when compared with all other methods over fivefolds using t-test at the 5% significance level is denoted by ^a^

**Fig. 2:**
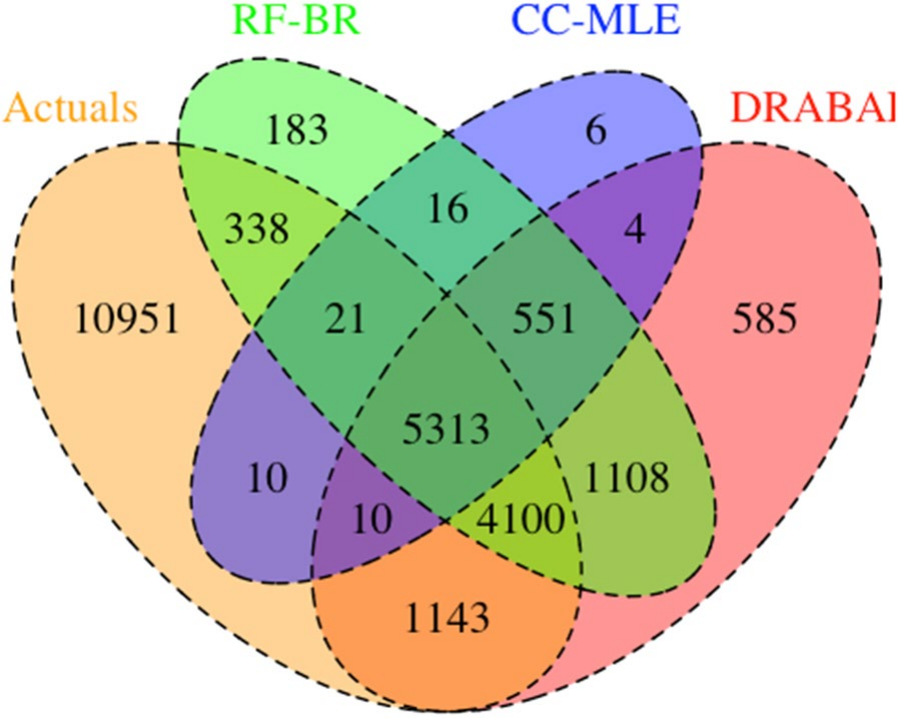
Venn diagram of correct predictions for four selected methods. The diagram includes average number of counts (i.e. average of fivefold cross-validation) of correct predictions using four methods and counts matching with actual ground truth

In Fig. 2, we analyze the effect of applying these methods over several datasets to see how many real positive interactions can be predicted correctly by the methods as absolute numbers. These absolute numbers translate to the number of actual positive experiments in the lab, which if predicted correctly means that the method is doing well in capturing the true nature of the positive interactions in these datasets. As shown in Fig. 2, we compared the absolute number of actual positive interactions (average size of 21,885 over fivefolds) to the number of positive predictions made by DRABAL, RF-BR (Second best preforming method), and CC-MLE (other variant of MLC solutions), which when applied to these datasets, averaged over a 5-cross validation setup. DRABAL predicted 10,566 real positive interactions correctly of which 1143 were uniquely identified by DRABAL. On the other hand, RF-BR predicted 9772 real positive interactions of which only 338 were unique to RF-BR, and CC-MLE correctly predicted 5354 of which only 10 were unique. Combined with previous summary results, we can conclude that DRABAL has identified the largest unique set while performing better in terms of GMean, F_1_Score and F_0.5_Score.

In this subsection, we evaluated the performance of DRABAL over five challenging large HTS assay datasets. In order to show that DRABAL is not limited to a specific number of datasets, we consider also an extended selection of ten BioAssays, and report a performance evaluation of over about 3 million interactions for 431,478 unique compounds (see Additional file 1: Table S1 and Table S2).

### Suggested drug-multi-target repositioning

In order to show the utility of DRABAL, we screened all the approved drugs from DrugBank database against assays used in this study. Table 3 shows the top five novel predictions for each assay. Interestingly, both Omeprazole (DB00338) and Thiabendazole (DB00730) are predictions for BioAssays AID 485297 and AID 485313. These BioAssays are two high-throughput assays for screening activators of Ras-related protein (Rab-9A) and a Niemann-Pick C1 protein (NPC1), respectively [46, 47]. However, DRABAL prediction scores show that Thiabendazole is the more likely activator of the Rab-9A and NPC1 proteins (see Table 3). When activators overexpress Rab-9A and NPC1, it was experimentally shown that the symptoms of the Niemann-Pick type C (NPC) disease are reduced [46, 47]. Thus, we will focus on the repurposing of Thiabendazole as a plausible treatment of the NPC disease.

**Table 3:** Top five predicted interactions from DrugBank approved drugs database

Rank	AID 1458 Target: Survival of motor neuron 2 (DRABAL score)	AID 485297 Target: Ras-related protein Rab-9A (DRABAL score)	AID 485313 Target: Niemann-Pick C1 protein precursor (DRABAL score)	AID 588342 Target: Luciferase transcriptional reporter (DRABAL score)	AID 686978 Target: Tyrosyl-DNA phosphodiesterase 1 (DRABAL score)
1	Amlexanox DB01025 (0.48)	Nitazoxanide DB00507 (0.67)	Thiabendazole DB00730 (0.33)	Phenazopyridine DB01438 (0.68)	Vinblastine DB00570 (0.99)
2	Mycophenolate mofetil DB00688 (0.3)	Thiabendazole DB00730 (0.61)	Omeprazole DB00338 (0.21)	Mitoxantrone DB01204 (0.53)	Plicamycin DB06810 (0.99)
3	Rabeprazole DB01129 (0.2)	Omeprazole DB00338 (0.23)	Phenazopyridine DB01438 (0.21)	Phenindione DB00498 (0.49)	Bromocriptine DB01200 (0.98)
4	Pramipexole DB00413 (0.14)	Nabumetone DB00461 (0.19)	Mebendazole DB00643 (0.12)	Olsalazine DB01250 (0.47)	Ketoconazole DB01026 (0.98)
5	Idoxuridine DB00249 (0.13)	Mycophenolic acid DB01024 (0.18)	Olsalazine DB01250 (0.12)	Amsacrine DB00276 (0.44)	Teniposide DB00444 (0.97)

Thiabendazole (DB00730) is the top common prediction for BioAssays AID 485297 and AID 485313

NPC disease is a rare neurodegenerative lipidosis associated with mutations that inactivate either NPC1 (95% of cases) or NPC2 proteins [48]. In healthy individuals, these proteins cooperate to aid the movement of unesterified cholesterol through the lysosome, to the cytosolic compartment of cells through the body [49]. Mutations that inactivate the NPC proteins cause endosomal/lysosomal accumulation of cholesterol, progressive neurodegeneration, and robust glial cell activation [50]. In NPC disease pathogenesis, glial cells such as astrocytes and microglia are activated and characterized with high concentrations of interleukin-6 (IL-6), cathepsin D, interferon-beta and interleukin-8 (IL-8), as well as signal transducers and activators of transcription (STATs) and TLR4 [51]. NPC disease is additionally characterized by increased Beclin-1 levels and elevated autophagy [52]. Taken together, impaired trafficking of cholesterol was further shown to mediate toxicity and increased cathepsin D levels that induce neurotoxicity by activating the autophagic pathway [53].

Our predicted activator, Thiabendazole is the drug of choice for strongyloidiasis and is originally used against a variety of nematodes [54]. It is an aryl hydrocarbon receptor ligand which has been shown to reduce levels of cathepsin D [55], overexpression of which is one of the characteristics of NPC disease. Also, it has been demonstrated that Thiabendazole is a potent inhibitor of cytochrome P450 1A2 (CYP1A2) [56], a major CYPs that metabolize drugs in the liver [57]. Additionally, cytochrome P450 proteins in general, have been shown to play different roles in the brain such as neuroprotection, neurotrophic support, temperature control, control of cerebral blood flow, maintenance of brain cholesterol homoeostasis, neuropeptide release, regulation of neurotransmitter levels, elimination of retinoids from CNS and other roles important in brain development, physiology and disease [58]. It has been reported that an ‘overdosage’ of Thiabendazole may be associated with psychic alterations and temporary vision disturbance [59]. With Thiabendazole therapy, the more common side effects include nausea, anorexia, diarrhea, dizziness, increased blood sugar levels and erythema multiforme [60]. These well-known reported side effects show that Thiabendazole has been extensively used in various therapies.

Thiabendazole, that we predict to activate both Rab-9A and NPC1 proteins, belongs to the Benzenoid superclass. We note that Benzoic Acid (DB03793) [61], an approved drug in DrugBank database belonging to the same Benzenoid superclass, was reported to target the Rab-9A protein. Also, note that the Ezetimibe drug (DB00973), having Benzenoid as one of its substituents, is reported to target the NPC1 protein leading to lowering cholesterol levels [62].

**Fig. 3:**
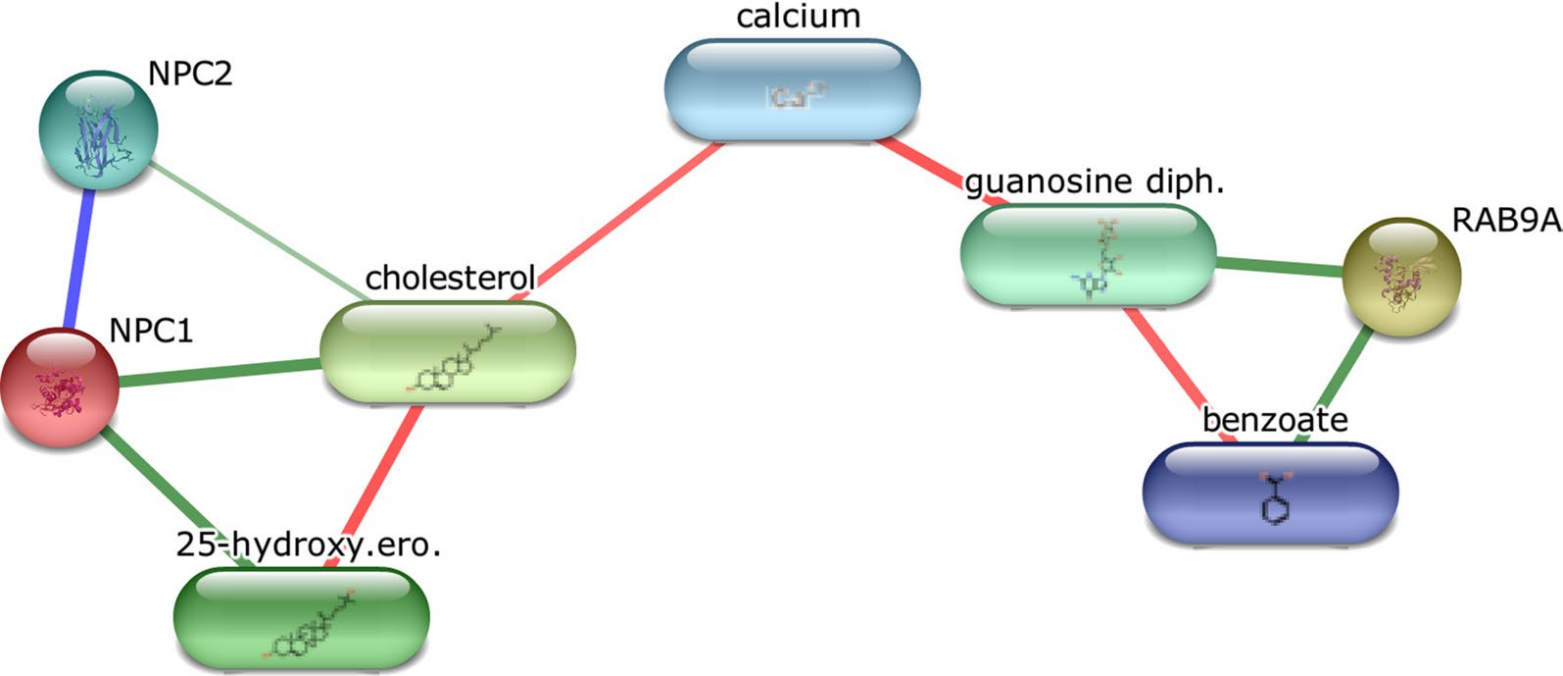
Chemical-Protein interactions graph generated using STITCH tool. STITCH tool was queried using NPC1 and Rab-9A concepts and then produced this graph. Nodes, which show concepts not directly related to this generated graph, were removed in order to highlight most relevant concepts to the repositioned drug

We used the STITCH database [63] to further query the relevant connections between Rab-9A and NPC1 and generated the graph in Fig. 3. When considering the interaction list connecting the two proteins, cholesterol and Benzoate, we find that Benzoate shares the same Benzenoid superclass as Thiabendazole, and is directly connected to Rab-9A. It is also interesting that calcium is the connecting hub because it has been demonstrated that for lysosomal exocytosis, VAMP7 (vesicle-associated membrane protein 7) on the surface of lysosomes, pulls and docks the lysosomes on the cytoplasmic side of the plasma membrane to form a trans-SNARE complex with syntaxin-4 and SNAP23 (synaptosome-associated protein of 23 kDa) on the plasma membrane [64], an action that is triggered by a rise in intracellular calcium levels [65, 66]. It should be noted that the VAMP7 is used by both NPC1 and Rab-9A associated lysosomal exocytosis. Additionally, even though the relationship between calcium and Thiabendazole has not been shown in humans, an increase in the fruit calcium content is used in the management of pear trees, as increased calcium content has been shown to reduce the severity of the decay and increase the efficacy of Thiabendazole when it is used as the postharvest fungicide [67]. Although the network does not show the character of interactions, STITCH listed concepts (benzoate, RAB9A, NPC1, calcium) which can in principle be linked to Thiabendazole. These findings add confidence to our suggestion that Thiabendazole may be an activator of both the Rab-9A and NPC1 protein, and thus suggest the repurposing of Thiabendazole to treat Niemann-Pick type C (NPC) disease.

## Conclusions

With the expansion and emergence of biomedical data and computational resources, there is a growing opportunity for impacting the process of drug repositioning and drug discovery. Many laboratory experiments have been developed to screen activities of chemical compounds over some biological targets. The ability to exploit feedback from these experiments can greatly enhance our decisions about cases, which were not tested for a particular biological target. Correlating feedback from different HTS assays, can improve our understanding about pathways of interactions. Motivated by these facts, we formulated the problem of virtual screening from high-throughput screening assays as a multi-label classification problem. This formulation allows us to model correlations and dependencies between the examined HTS assays and enhance prediction performance. The main challenge we face is that these assays do not report interactions for all compounds and thus, we have to handle the issue of missing labels. We developed a novel solution based on a Bayesian active learning framework to overcome this challenge and exploit actual dependencies between the HTS assays. Compared to the other state-of-the-art MLC methods, our proposed solution DRABAL improves the F_1_Score significantly by about 22% in absolute measures, on average. We also enable drug-multi-target repositioning and suggest the Thiabendazole drug as both a NCP1 and RAB-9A promoter activator, making it a possible treatment modality for Niemann–Pick type C disease.

## Methods

### Experimental data

#### PubChem BioAssay Database

We used confirmatory HTS assays from the PubChem BioAssay Database following recommendations of [68]. A BioAssay dataset is a report of a laboratory experiment, where the activity status of selected chemical compounds, with regard to a specific biological target, is listed. We chose five BioAssays that share a larger number of common active compounds in order to test the applicability of multi-label learning. For retrieving such related BioAssays, we first downloaded the largest high-throughput screening assay from the PubChem BioAssay Database [38]. The examined datasets belong to the confirmatory experiments over protein targets that were deposited by the NIH Molecular Libraries Program. Some BioAssays hold a very large number of interactions but with only an extremely small set of active cases. For example, the BioAssay record for AID 602332 holds a total of 424,929 interactions with only 77 active cases (active/inactive imbalance ratio is 77/424,929 = 0.01%). These BioAssays were excluded from the initial selection list. After selecting the largest HTS assay (i.e. AID 588342), based on these conditions, we retrieved the four other mostly related BioAssays to this one in terms of common active compounds. Finally, we ended up with a total of five datasets as summarized in Table 4. In another set of experiments, we consider ten datasets to show that DRABAL is not limited by a certain number of assigned target labels (see Additional file 1: Table S1 and Table S2).

**Table 4:** Summary of datasets used

Dataset PubChem ID	Target name	Type of interacting compounds	Active class size	Inactive class size	Active to inactive ratio (imbalance ratio)
AID 1458	Survival of motor neuron 2	Enhancers	5854	193,105	1:33
AID 485297	Ras-related protein Rab-9A	Activators	9143	301,951	1:33
AID 485313	Niemann-Pick C1 protein precursor	Activators	7586	304,846	1:40
AID 588342	Luciferase transcriptional reporter	Inhibitors	25,159	304,600	1:12
AID 686978	Tyrosyl-DNA phosphodiesterase 1	Inhibitors	64,212	243,136	1:4
Total interactions			1,459,592		

Among the five selected HTS assays, the percentage of common active interactions is around 37% on average. For each BioAssay dataset, a positive label ‘+1’ indicates that the compound is active in the assay, while a negative ‘−1’ relates to inactive compounds. An inactive compound, although indicates a negative outcome under the examined assay setup, may relate to another phenotype of interaction with the biological target. For the MLC setup, assays are integrated such that a single record about a chemical compound would hold all its relevant interactions in the examined BioAssays. Given this setup, a missing label with a value of ‘0’ is assigned for each compound that does not have a reported activity status in a particular assay. While compiling and extracting features for compounds, a Cheminformatics toolkit used failed to generate part of the features in few cases. This happens when the compound’s input file did not contain sufficient details needed by the Cheminformatics toolkit to compile and produce all required information. We excluded such compounds. After the data-cleaning step, we ended up with 411,112 unique chemical compounds for all the datasets. These five datasets hold 1,448,403 interactions with only around an 8% hit rate indicating positive interaction cases with the targets. Our target matrix is sparse with around 30% missing labels, providing the chance for about 600,000 potential novel interactions. To the best of our knowledge, this is the largest compiled dataset for a virtual screening study on HTS assays from the PubChem BioAssay Database. Table 4 summarizes basic information of the datasets we used.

#### DrugBank

We downloaded DrugBank database data in February, 2016 from http://www.drugbank.ca/ [69]. The database contained 7097 drug entries including 1826 FDA-approved drugs. We only used FDA-approved drugs to screen by models we developed for the HTS assays.

### Feature generation and selection

The generation and selection of a representative subset of features is critical for developing an accurate classification model [70]. A wide variety of chemical features have been proposed for models used for virtual screening [68, 71]. For our study, we combined fingerprint features generated by OpenBabel [72] and RDKit [73], including PubChem fingerprints [74]. We computed several types of features such as the number of H-acceptors and donors, molecular weight, and Log-P, etc. The final set contained 2940 features. With such a large set of compiled features, there is a higher chance of different levels of information redundancy, and it may contain also features not related to the types of biological activity of chemicals, as observed in particular HTS assays. Thus, we follow a feature selection (FS) procedure, similar to the one we have suggested in an earlier work [12]. For optimizing the selection of a subset of relevant features, the DWFS tool was used [75]. A detailed description of 1064 features selected and used in the study is provided in Additional file 3.

### Classifiers

To compare alternative MLC solutions for activity screening in PubChem HTS assays we used three types of classifiers. These include support vector machines (SVM) [76] with radial basis function (RBF) kernels, K-nearest neighbors (KNN; K = 3) [77], and Random Forests (RF; trees = 500) [78]. The RBF kernel widths and default value of the cost parameter were used for SVM. Calling the algorithms was done using the Scikit-learn machine learning package [79, 80]. We used a cluster of Linux based machines with 64 cores and 256 GB RAM per node for processing the data and running the experiments.

### Bayesian network structure learning

For learning the corresponding BN structure for the generated data, we used the BN structure learning, from the discrete data algorithm that was implemented in the libPGM package [81].

### Methods

#### Existing multi-label classification (MLC) methods for virtual screening

HTS assays report experimental outcomes of testing different biological activities of chemical compounds. Shared common activities between these assays can enhance our understanding of the pathways of interactions especially when it is difficult to infer an explicit relationship between the biological targets (e.g. lacking protein–protein interaction or lacking sequence similarity). MLC methods directly address this motivation through exploiting existing dependencies between the examined HTS assays. Many modern applications in fact, also require this formulation such as classification of protein functions and semantic scenes [26].

Traditional single-label classification learns from a set of cases, each associated with a single unique label from a set $L$, $\left| L \right| > 1$ [22]. When $\left| L \right| = 2$, it refers to a binary classification, and if $\left| L \right| > 2$, it refers to a multi-class classification. However, the MLC classification task refers to a set of cases each associated with a set of labels $Y \subseteq L$ and not a unique label. Thus, instead of assigning a scalar output for a sample, MLC assigns a vector indicating the corresponding group of assigned labels. MLC classification methods can be grouped into: (a) problem transformation methods, and (b) algorithm adaptation methods [25]. The methods in the first group are independent of the learning algorithm and suggest transformation of MLC learning task into simpler tasks that any classifier can deal with. The other group represents a class of methods based on extending learning algorithms for MLC data, like multi-label artificial neural networks [82]. Given our interest in exploring the novel application of MLC with flexibly any type of classifier, we focus on problem transformation methods.

A conventional MLC transformation solution is based on independently training a single binary classifier for each target label. For each new instance, the trained models are used to assign a set of labels where the instance is predicted as a positive. This baseline approach is known as binary relevance (BR), and in general, it is quite an accurate approach [34, 35]. In order to model label correlations with a chain of binary classifiers, the classifier chains (CC) approach was introduced [36]. This method, which showed performance improvements in particular scenarios, is based on training classifiers such that the training data for each classifier is extended by including the target labels of the previous one, which would in a way resemble a chain order [36, 83]. The order of classifiers is initialized randomly. In the context of HTS assays, the target label set of a particular assay is just considered as an extra-added feature for another assay that follows the chain order. Given this formulation, the labels of a particular dataset cannot be added as a feature to another one if there is a difference in terms of the number of training samples of each. In other words, missing labels for one dataset needs to be addressed before including it as a new feature for another different size dataset. Thus, we replace missing labels with negative labels and extend CC to classifier chains with a missing labels extension (CC-MLE). Treating missing labels as negative labels is one of the approaches to handle missing labels in MLC problems [45], as well as because negative labels in many cases reflect the majority of all target labels for the examined HTS assays that happen to be inactive in the assay. For DRABAL, however, we handle missing labels differently using active learning which helps in quantifying a probability score of interaction for each missing case instead of assuming it negative. In general, once trained, classifiers return probabilities of input samples to be members of the positive or negative classes. For example, a sample that has two positive nearest neighbors out of three neighbors, KNN (K = 3) classifier returns a probability score of 0.67. In this way, the returned value can be used to quantify the score (i.e. the probability score for a sample to be a member of the positive class). For samples with missing target labels, these scores can be used to replace the missing values. Since we train the model and then use its feedback for other samples with missing labels, we consider the setup to follow active learning approach.

Other than how DRABAL handles missing labels, there are two more differences relative to CC-MLE. CC-MLE will generate different outcomes depending on the order of the labels used to extend the feature sets. DRABAL, on the other hand, using the Bayesian network determines a specific order of labels to extend feature sets, while satisfying existing dependencies between the target labels. Also, CC-MLE adds one feature to the second model, two features to the third model, and continues until $\left| {\text{L}} \right| - 1$ features are added to the final model, where $\left| {\text{L}} \right|$ is the total number of target labels. DRABAL adds features based on dependency and thus, for any model, any number of features can be added and, for example, it is not necessary that the last model will have $\left| {\text{L}} \right| - 1$ added features. In addition to differences of DRABAL and CC, we point out that the main differences between DRABAL and BR-RF. BR-RF does not address potential correlation between target class labels in any manner. Instead, DRABAL exploits existing relationships between the labels and incorporate them as part of the training of the classifiers.

While on one hand we seek to model correlation or dependency between the HTS assays, we lack a considerable amount of information about activities of compounds that were not reported in a particular assay. Some compounds that were reported as either active or inactive in a specific HTS assay were not tested in other ones. This type of missing information imposes a challenge for the MLC classification task. Although in recent years MLC has gained a noticeable amount of interest, most of the existing approaches do not adequately address the ability to handle data with missing labels [34]. Very recently, there have been several studies on proposing MLC algorithms that can directly deal with missing label problems [34, 45, 84, 85]. These methods are not necessarily problem transformation methods, where state-of-the-art existing classifiers can be used and sometimes require extra information a domain-expert may need to provide, as in [85]. In our work, we present a novel problem transformation method (i.e. one suitable for a wide variety of classifiers) that can handle missing labels for MLC problems.

#### DRABAL: our proposed solution

**Fig. 4:**
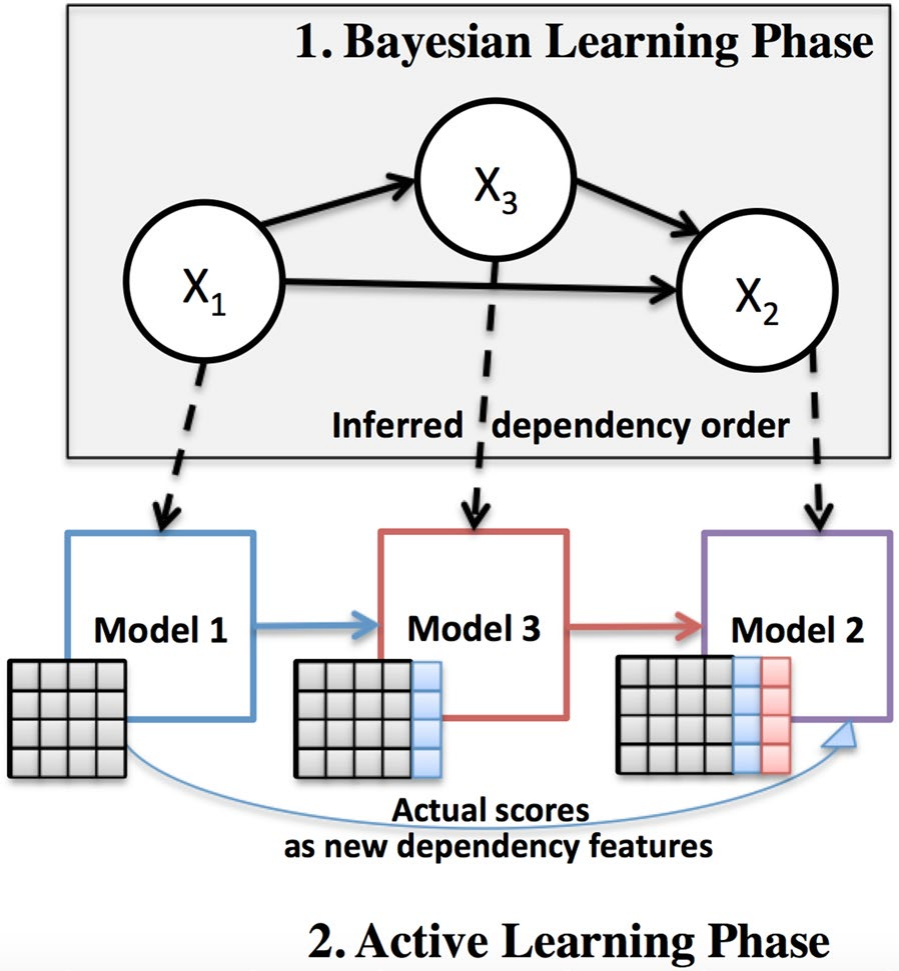
Illustration of our proposed method DRABAL. DRABAL has two learning phases including a Bayesian learning phase and an active learning phase for building the multi-label classification models

DRABAL is a novel problem transformation MLC solution, based on inferring dependencies and handling missing labels. As illustrated in Fig. 4, DRABAL has two learning phases including a Bayesian learning phase and an active learning phase for building the MLC models.

#### Bayesian learning phase: learning conditional dependencies between HTS assays

For the first phase, we learn the full structure of a Bayesian network (BN) that models dependencies between the discrete target labels of the HTS assays. BN is a probabilistic graphical model that represents a set of random variables and conditional dependencies, among them using a directed acyclic graph (DAG). Instead of randomly assuming the relationships between the target classes in the MLC setup like in CC, BN properly defines all relevant conditional dependencies. For learning the structure of the BN, the pairwise conditional independencies between the target labels are tested. For two target labels $y_{i}$ and $y_{j}$, they are considered conditionally independent if Eq. (7) holds.

7 $$P\left( {y_{i} , y_{j} |\bar{Y}} \right) = P\left( {y_{i} |\bar{Y}} \right) \times P\left( {y_{j} |\bar{Y}} \right) ; \bar{Y} = Y - \left\{ {y_{i} , y_{j} } \right\}$$

**Fig. 5:**
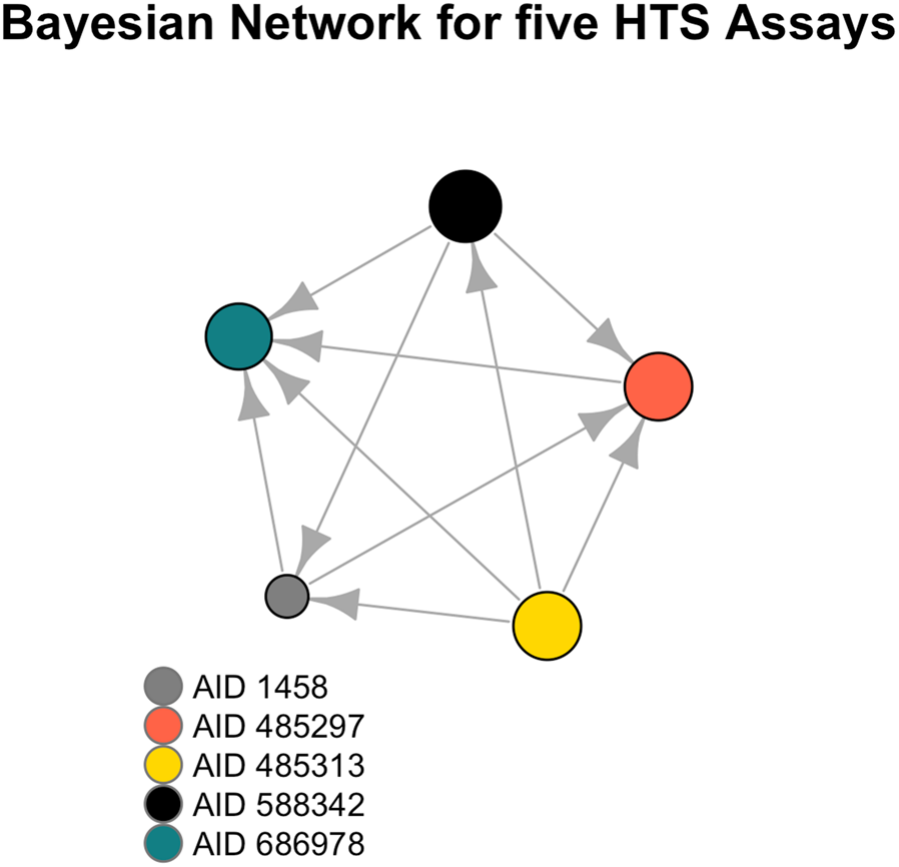
Bayesian network for five used HTS assays. Size of the node indicates the number of positive interactions reported in the corresponding HTS assay

Once conditional independence is computed between every pair of HTS assays, a DAG is built using a Build-PDAG algorithm [86]. Given this representation, nodes represent target labels of HTS assays and edges correspond to the direct influence that assays would have on one another. Figure 5 illustrates the BN structure we learned for the examined HTS assays, from the PubChem BioAssay Database. Given a classifier $C_{l}$ learned for an assay $l$ and $pa\left( {C_{l} } \right)$ as the set of parents of the classifier $C_{l}$ as inferred by the BN, the probability for a chemical compound $x_{k}$ to be active (i.e. label value is ‘1’) is defined as in Equation [8].

8 $$p\left( {C_{l} = 1 |x_{k} } \right) = p\left( {C_{l} = 1 |pa\left( {C_{l} } \right) = 1, x_{k} } \right)$$

As an example, for AID 1458 HTS assay (see Fig. 5), $p\left( {C_{AID 1458} = 1 |x_{k} } \right)$ is expressed as in Equation [9].

9 $$p\left( {C_{AID 1458} = 1 |x_{k} } \right) = p\left( {C_{AID 1458} = 1 |C_{AID 485313} = 1, C_{AID 588342} = 1, x_{k} } \right)$$

Intuitively, a chemical compound that is active in both AID 485313 and AID 588342 assays, affects the decision of whether it is active or not in AID 1458.

#### Active learning phase: employing classifier feedback as dependency features

After learning the BN structure, we topologically sort the nodes of the graph and then, start building a classifier for each node in this order (see Fig. 4). Active learning (AL), is based on the idea of establishing a feedback loop between the training set and the classifier to improve prediction performance [87]. Motivated by this idea, we use the actual output scores of the learning algorithms (or classifiers) as a type of new feature to be shared based on the dependency structure inferred in the first phase. For the previously given example, classifiers are trained for assays AID 485313 and AID 588342. The probability scores for compounds to be active based on these classifiers are then shared (i.e. added as extra features to training set) with AID 1458. There upon, a classifier for AID 1458 can be trained based on this feedback information, propagated from its parents in the BN. This type of shared information between every classifier and its parents in the BN structure, emulates an active learning step. An intrinsic advantage of this type of learning for MLC, is that it can alleviate the problem of a poor classifier which will make erratic predictions and consequently affect the subsequent classifiers [88]. Since we also utilize this feedback from the classifiers, we can easily replace missing labels with probability scores a classifier assigns after training.

Once all models are trained based on the DRABAL framework, for each new testing instance, all classifiers should be applied following the topological order of the BN. For any new instance, after the classifier gives a decision on its type of activity, its probability of being positive is propagated to children nodes (i.e. dependent classifiers) of the network. Finally, every classifier will predict the decision over this new instance given the shared knowledge from other classifiers. Pseudocode of DRABAL is given in Additional file 4.

## Additional files

**Additional file 1.**Performance comparison of all methods over ten large HTS assays composed of 3 million interactions for 431,478 unique compounds from PubChem BioAssay Database.

**Additional file 2.**Extended performance comparison using different validation methods. Holdout with training proportions from 80 to 20% are used and external samples are generated for more challenging tests.

**Additional file 3.**Summary description of selected and generated features for chemical compounds.

**Additional file 4.**Pseudocode of DRABAL.

## References

[1] Jensen PB, Jensen LJ, Brunak S (2012). Mining electronic health records: towards better research applications and clinical care. Nat Rev Genet.

[2] Burke W, Burton H, Hall AE, Karmali M, Khoury MJ, Knoppers B (2010). Extending the reach of public health genomics: what should be the agenda for public health in an era of genome-based and “personalized” medicine?. Genet Med.

[3] Macarron R, Banks MN, Bojanic D, Burns DJ, Cirovic DA, Garyantes T (2011). Impact of high-throughput screening in biomedical research. Nat Rev Drug Discov.

[4] Paul SM, Mytelka DS, Dunwiddie CT, Persinger CC, Munos BH, Lindborg SR (2010). How to improve R&D productivity: the pharmaceutical industry’s grand challenge. Nat Rev Drug Discov.

[5] Pardalos PM, Boginski VL, Alkis V (2008). Data mining in biomedicine.

[6] Shoichet BK (2004). Virtual screening of chemical libraries. Nature.

[7] Reutlinger M, Koch CP, Reker D, Todoroff N, Schneider P, Rodrigues T (2013). Chemically advanced template search (CATS) for scaffold-hopping and prospective target prediction for ‘orphan’ molecules. Mol Inform.

[8] Nidhi A, Glick M, Davies JW, Jenkins JL (2006). Prediction of biological targets for compounds using multiple-category Bayesian models trained on chemogenomics databases. J Chem Inf Model.

[9] Mervin LH, Afzal AM, Drakakis G, Lewis R, Engkvist O, Bender A (2015). Target prediction utilising negative bioactivity data covering large chemical space. J Cheminform.

[10] Keiser MJ, Setola V, Irwin JJ, Laggner C, Abbas AI, Hufeisen SJ (2009). Predicting new molecular targets for known drugs. Nature.

[11] Lo YC, Senese S, Damoiseaux R, Torres JZ (2016). 3D chemical similarity networks for structure-based target prediction and scaffold hopping. ACS Chem Biol.

[12] Soufan O, Ba-Alawi W, Afeef M, Essack M, Rodionov V, Kalnis P (2015). Mining chemical activity status from high-throughput screening assays. PLoS ONE.

[13] Munkhdalai T, Li M, Batsuren K, Park HA, Choi NH, Ryu KH (2015). Incorporating domain knowledge in chemical and biomedical named entity recognition with word representations. J Cheminformatics.

[14] Ba-Alawi W, Soufan O, Essack M, Kalnis P, Bajic VB (2016). DASPfind: new efficient method to predict drug-target interactions. J Cheminform.

[15] Webb SJ, Hanser T, Howlin B, Krause P, Vessey JD (2014). Feature combination networks for the interpretation of statistical machine learning models: application to Ames mutagenicity. J Cheminform.

[16] Schneidman-Duhovny D, Nussinov R, Wolfson HJ (2004). Predicting molecular interactions in silico: II. Protein–protein and protein–drug docking. Curr Med Chem.

[17] Xie XQ, Chen JZ (2008). Data mining a small molecule drug screening representative subset from NIH PubChem. J Chem Inf Model.

[18] Wang X, Chen H, Yang F, Gong J, Li S, Pei J (2014). iDrug: a web-accessible and interactive drug discovery and design platform. J Cheminform.

[19] Liu X, Vogt I, Haque T, Campillos M (2013). HitPick: a web server for hit identification and target prediction of chemical screenings. Bioinformatics.

[20] Sakakibara Y, Hachiya T, Uchida M, Nagamine N, Sugawara Y, Yokota M (2012). COPICAT: a software system for predicting interactions between proteins and chemical compounds. Bioinformatics.

[21] Kuhn M, von Mering C, Campillos M, Jensen LJ, Bork P (2008). STITCH: interaction networks of chemicals and proteins. Nucleic Acids Res.

[22] Katakis I, Tsoumakas G, Vlahavas I (2008) Multilabel text classification for automated tag suggestion. ECML PKDD discovery challenge 75

[23] Wang H, Huang H, Ding C (2009) Image annotation using multi-label correlated green’s function. In: 2009 IEEE 12th International Conference on Computer Vision. IEEE, pp 2029–2034​

[24] Cheng W, Hüllermeier E (2009). Combining instance-based learning and logistic regression for multilabel classification. Mach Learn.

[25] Zhang M-L, Zhou Z-H (2014). A review on multi-label learning algorithms. IEEE Trans Knowl Data Eng.

[26] Tsoumakas G, Katakis I (2006). Multi-label classification: an overview.

[27] Wang X, Zhang W, Zhang Q, Li GZ (2015). MultiP-SChlo: multi-label protein subchloroplast localization prediction with Chou’s pseudo amino acid composition and a novel multi-label classifier. Bioinformatics.

[28] Gonen M, Margolin AA (2014). Drug susceptibility prediction against a panel of drugs using kernelized Bayesian multitask learning. Bioinformatics.

[29] Heider D, Senge R, Cheng W, Hullermeier E (2013). Multilabel classification for exploiting cross-resistance information in HIV-1 drug resistance prediction. Bioinformatics.

[30] Michielan L, Terfloth L, Gasteiger J, Moro S (2009). Comparison of multilabel and single-label classification applied to the prediction of the isoform specificity of cytochrome p450 substrates. J Chem Inf Model.

[31] Afzal AM, Mussa HY, Turner RE, Bender A, Glen RC (2015). A multi-label approach to target prediction taking ligand promiscuity into account. J Cheminform.

[32] Dahl GE, Jaitly N, Salakhutdinov R (2014) Multi-task neural networks for QSAR predictions. arXiv preprint arXiv:14061231

[33] Unterthiner T, Mayr A, Klambauer G, Hochreiter S (2015) Toxicity prediction using deep learning. arXiv preprint arXiv:150301445

[34] Yu H-F, Jain P, Kar P, Dhillon IS (2013) Large-scale multi-label learning with missing labels. arXiv preprint arXiv:13075101

[35] Fürnkranz J, Hüllermeier E, Mencía EL, Brinker K (2008). Multilabel classification via calibrated label ranking. Mach Learn.

[36] Read J, Pfahringer B, Holmes G, Frank E (2011). Classifier chains for multi-label classification. Mach Learn.

[37] Wu B, Lyu S, Ghanem B. Constrained submodular minimization for missing labels and class imbalance in multi-label learning. In: Proceedings of the thirtieth AAAI conference on artificial intelligence (AAAI-16), pp 2229–2236

[38] Wang Y, Xiao J, Suzek TO, Zhang J, Wang J, Bryant SH (2009). PubChem: a public information system for analyzing bioactivities of small molecules. Nucleic Acids Res.

[39] Wishart DS, Knox C, Guo AC, Shrivastava S, Hassanali M, Stothard P (2006). DrugBank: a comprehensive resource for in silico drug discovery and exploration. Nucleic Acids Res.

[40] He H, Garcia EA (2009). Learning from imbalanced data. IEEE Trans Knowl Data Eng.

[41] Maitin-Shepard J, Cusumano-Towner M, Lei J, Abbeel P (eds) (2010) Cloth grasp point detection based on multiple-view geometric cues with application to robotic towel folding. In: 2010 IEEE international conference on robotics and automation (ICRA), IEEE

[42] Santoni FA, Hartley O, Luban J (2010). Deciphering the code for retroviral integration target site selection. PLoS Comput Biol.

[43] Braga-Neto UM, Dougherty ER (2004). Is cross-validation valid for small-sample microarray classification?. Bioinformatics.

[44] Spyromitros E, Tsoumakas G, Vlahavas I (2008). An empirical study of lazy multilabel classification algorithms. Artificial intelligence: theories, models and applications.

[45] Wu B, Liu Z, Wang S, Hu B-G, Ji Q (eds) (2014) Multi-label learning with missing labels. In: 2014 22nd International conference on pattern recognition (ICPR), IEEE

[46] Information NCfB. PubChem BioAssay database AID 485313. https://pubchem.ncbi.nlm.nih.gov/bioassay/485313

[47] Information NCfB. PubChem BioAssay database AID 485297. https://pubchem.ncbi.nlm.nih.gov/bioassay/485297

[48] Xu M, Liu K, Swaroop M, Porter FD, Sidhu R, Firnkes S (2012). delta-Tocopherol reduces lipid accumulation in Niemann-Pick type C1 and Wolman cholesterol storage disorders. J Biol Chem.

[49] Blanchette-Mackie EJ (2000). Intracellular cholesterol trafficking: role of the NPC1 protein. Biochim Biophys Acta.

[50] Suzuki M, Sugimoto Y, Ohsaki Y, Ueno M, Kato S, Kitamura Y (2007). Endosomal accumulation of Toll-like receptor 4 causes constitutive secretion of cytokines and activation of signal transducers and activators of transcription in Niemann-Pick disease type C (NPC) fibroblasts: a potential basis for glial cell activation in the NPC brain. J Neurosci.

[51] German DC, Liang CL, Song T, Yazdani U, Xie C, Dietschy JM (2002). Neurodegeneration in the Niemann-Pick C mouse: glial involvement. Neuroscience.

[52] Pacheco CD, Lieberman AP (2007). Lipid trafficking defects increase Beclin-1 and activate autophagy in Niemann-Pick type C disease. Autophagy.

[53] Amritraj A, Wang Y, Revett TJ, Vergote D, Westaway D, Kar S (2013). Role of cathepsin D in U18666A-induced neuronal cell death: potential implication in Niemann-Pick type C disease pathogenesis. J Biol Chem.

[54] DrugBank. DB00730 Thiabendazole. http://www.drugbank.ca/drugs/DB00730

[55] Ramadoss P, Marcus C, Perdew GH (2005). Role of the aryl hydrocarbon receptor in drug metabolism. Expert Opin Drug Metab Toxicol.

[56] Bapiro TE, Sayi J, Hasler JA, Jande M, Rimoy G, Masselle A (2005). Artemisinin and thiabendazole are potent inhibitors of cytochrome P450 1A2 (CYP1A2) activity in humans. Eur J Clin Pharmacol.

[57] Wang B, Zhou SF (2009). Synthetic and natural compounds that interact with human cytochrome P450 1A2 and implications in drug development. Curr Med Chem.

[58] Liu M, Hurn PD, Alkayed NJ (2004). Cytochrome P450 in neurological disease. Curr Drug Metab.

[59] DrugLib.com. Thiabendazole. http://www.druglib.com/activeingredient/thiabendazole/

[60] Drugs.com. Thiabendazole Side Effects. http://www.drugs.com/sfx/thiabendazole-side-effects.html

[61] DrugBank. DB03793 Benzoic Acid. http://www.drugbank.ca/drugs/DB03793

[62] DrugBank. DB00973 Ezetimibe. http://www.drugbank.ca/drugs/DB00973

[63] Kuhn M, Szklarczyk D, Pletscher-Frankild S, Blicher TH, von Mering C, Jensen LJ (2014). STITCH 4: integration of protein-chemical interactions with user data. Nucleic Acids Res.

[64] Rao SK, Huynh C, Proux-Gillardeaux V, Galli T, Andrews NW (2004). Identification of SNAREs involved in synaptotagmin VII-regulated lysosomal exocytosis. J Biol Chem.

[65] Rodriguez A, Webster P, Ortego J, Andrews NW (1997). Lysosomes behave as Ca2+-regulated exocytic vesicles in fibroblasts and epithelial cells. J Cell Biol.

[66] Reddy A, Caler EV, Andrews NW (2001). Plasma membrane repair is mediated by Ca(2+)-regulated exocytosis of lysosomes. Cell.

[67] Sugar D, Basile SR (2011). Orchard calcium and fungicide treatments mitigate effects of delayed postharvest fungicide applications for control of postharvest decay of pear fruit. Postharvest Biol Technol.

[68] Schierz AC (2009). Virtual screening of bioassay data. J Cheminform.

[69] Wishart DS, Knox C, Guo AC, Shrivastava S, Hassanali M, Stothard P (2006). DrugBank: a comprehensive resource for in silico drug discovery and exploration. Nucleic Acids Res.

[70] Guyon I, Elisseeff A (2003). An introduction to variable and feature selection. J Mach Learn Res.

[71] Kong X, Yu PS (eds) (2010) Semi-supervised feature selection for graph classification. In: Proceedings of the 16th ACM SIGKDD international conference on knowledge discovery and data mining, ACM

[72] O’Boyle NM, Banck M, James CA, Morley C, Vandermeersch T, Hutchison GR (2011). Open Babel: an open chemical toolbox. J Cheminform.

[73] Landrum G (2006) RDKit: Open-source cheminformatics. Open source software: RDKit. Retrieved from: http://www.rdkit.org. Accessed 4 Mar 2012

[74] PubChem. PubChem substructure fingerprint 2009. ftp://ftp.ncbi.nlm.nih.gov/pubchem/specifications/pubchem_fingerprints.txt

[75] Soufan O, Kleftogiannis D, Kalnis P, Bajic VB (2015). DWFS: a wrapper feature selection tool based on a parallel genetic algorithm. PLoS ONE.

[76] Boser BE, Guyon IM, Vapnik VN (eds) (1992) A training algorithm for optimal margin classifiers. In: Proceedings of the fifth annual workshop on Computational learning theory, ACM

[77] Cover TM, Hart PE (1967). Nearest neighbor pattern classification. IEEE Trans Inf Theory.

[78] Breiman L (2001). Random forests. Mach Learn.

[79] Pedregosa F, Varoquaux G, Gramfort A, Michel V, Thirion B, Grisel O (2011). Scikit-learn: machine learning in Python. J Mach Learn Res.

[80] Buitinck L, Louppe G, Blondel M, Pedregosa F, Mueller A, Grisel O et al (2013) API design for machine learning software: experiences from the scikit-learn project. arXiv preprint arXiv:13090238

[81] Cabot C, Ulrich J, Raugas M (2012) A library for creating and using probabilistic graphical models libpgm 1.3. 2012. Open source software: libpgm 1.3; 2012. Retrieved from https://pypi.python.org/pypi/libpgm

[82] Zhang M-L, Zhou Z-H (2006). Multilabel neural networks with applications to functional genomics and text categorization. IEEE Trans Knowl Data Eng.

[83] Read J (2010). Scalable multi-label classification.

[84] Wu B, Lyu S, Hu B-G, Ji Q (2015). Multi-label learning with missing labels for image annotation and facial action unit recognition. Pattern Recogn.

[85] Wu B, Lyu S, Ghanem B (eds) (2015) ML-MG: multi-label learning with missing labels using a mixed graph. In: Proceedings of the IEEE international conference on computer vision

[86] Koller D, Friedman N (2009). Probabilistic graphical models: principles and techniques.

[87] Settles B (2010). Active learning literature survey. Univ Wis Madison.

[88] Sucar LE, Bielza C, Morales EF, Hernandez-Leal P, Zaragoza JH, Larrañaga P (2014). Multi-label classification with Bayesian network-based chain classifiers. Pattern Recogn Lett.

