# Supplementary material for: DRABAL: novel method to mine large high-throughput screening assays using Bayesian active learning
Source: J Cheminform. 2016 Nov 10;8:64. doi: 10.1186/s13321-016-0177-8 (PMC5105261; doi:10.1186/s13321-016-0177-8)
Supplement: Supplementary file 1 — Additional file 1.Performance comparison of all methods over ten large HTS assays composed of 3 million interactions for 431,478 unique compounds from PubChem BioAssay Database. [file 13321_2016_177_MOESM1_ESM.docx]

**Additional File 1**

**DRABAL: Novel Method for Mining Large High-throughput Screening Assays using Bayesian Active Learning**

**Othman Soufan^1^, Wail Ba-alawi^1^, Moataz Afeef^1^, Magbubah Essack^1^, Panos Kalnis^2^ and Vladimir B. Bajic^1,*^**

^1^King Abdullah University of Science and Technology (KAUST), Computational Bioscience Research Center (CBRC), Thuwal 23955-6900, Saudi Arabia.

^2^King Abdullah University of Science and Technology (KAUST), Infocloud Group, Computer, Electrical and Mathematical Sciences and Engineering Division (CEMSE), Thuwal 23955-6900, Saudi Arabia.

Author Emails:

Othman Soufan: othman.soufan@kaust.edu.sa,

Wail Ba-alawi: wail.baalawi@kaust.edu.sa,

Moataz Afeef: moataz.afeef@kaust.edu.sa,

Magbubah Essack: magbubah.essack@kaust.edu.sa,

Panos Kalnis: panos.kalnis@kaust.edu.sa,

Vladimir B. Bajic: vladimir.bajic@kaust.edu.sa

* Corresponding author: Vladimir B. Bajic: vladimir.bajic@kaust.edu.sa

Table S1 shows a summary of the 5-fold comparison results for ten HTS assays. Based on all summary evaluation metrics, DRABAL significantly outperformed other state-of-the-art methods. These results show similar improvements when five HTS assays are also used as reported in the main manuscript.

Table S1: Comparison between methods over ten different datasets based on 5-fold cross validation. These datasets include more than 3 million interactions for 431,478 unique compounds.

| **Method** | **GMean** | **F_1_Score** | **F_0.5_Score** |
| --- | --- | --- | --- |
| BR-SVM | 41.34% | 25.02% | 30.91% |
| BR-KNN | 22.34% | 13.22% | 21.85% |
| BR-RF | 51.68% | 41.43% | 55.45% |
| CC-MLE | 40.28% | 28.95% | 45.28% |
| DRABAL | **56.98%*** | **46.11%*** | **57.61%*** |

^*^ Indicates statistically significant difference when compared with all other methods over 5-folds using t-test at the 5% significance level.

Table S2 provides a summary of the ten datasets used in this set of experiments.

Table S2. Summary of experimental datasets including reference IDs in PubChem Database.

| **Dataset** | **Target Name** | **Type of interacting compounds** | **Active class size** | | **Inactive class size** | **Active to inactive ratio (Imbalance ratio)** |
| --- | --- | --- | --- | --- | --- | --- |
| AID 1458 | Survival of motor neuron 2 | Enhancers | 5,854 | | 193,105 | 1:33 |
| AID 485297 | Ras-related protein Rab-9A | Activators | 9,143 | | 301,951 | 1:33 |
| AID 485313 | Niemann-Pick C1 protein precursor | Activators | 7,586 | | 304,846 | 1:40 |
| AID 588342 | Luciferase transcriptional reporter | Inhibitors | 25,159 | | 304,600 | 1:12 |
| AID 686978 | Tyrosyl-DNA phosphodiesterase 1 | Inhibitors | 64,212 | | 243,136 | 1:4 |
| AID 686979 | Tyrosyl-DNA-phosphodiesterase I (TDP1) | Inhibitors | 49,946 | 264,132 | | 1:5 |
| AID 504466 | ATAD5 - ATPase family, AAA domain containing 5 | Inhibitors | 4,174 | 306,924 | | 1:73 |
| AID 504332 | Euchromatic histone-lysine N-methyltransferase 2 | Inhibitors | 31,109 | 270,505 | | 1:9 |
| AID 2551 | Nuclear receptor ROR-gamma | Inhibitors | 16,824 | 256,777 | | 1:15 |
| AID 624202 | BRCA1 - breast cancer 1 | Activators | 3,980 | 364,035 | | 1:91 |
| Total Interactions |  |  | 3,027,998 | | |  |
