# Supplementary material for: DRABAL: novel method to mine large high-throughput screening assays using Bayesian active learning
Source: J Cheminform. 2016 Nov 10;8:64. doi: 10.1186/s13321-016-0177-8 (PMC5105261; doi:10.1186/s13321-016-0177-8)
Supplement: Supplementary file 2 — Additional file 2.Extended performance comparison using different validation methods. Holdout with training proportions from 80 to 20% are used and external samples are generated for more challenging tests. [file 13321_2016_177_MOESM2_ESM.docx]

**Additional File 2**

**DRABAL: Novel Method for Mining Large High-throughput Screening Assays using Bayesian Active Learning**

**Othman Soufan^1^, Wail Ba-alawi^1^, Moataz Afeef^1^, Magbubah Essack^1^, Panos Kalnis^2^ and Vladimir B. Bajic^1,*^**

^1^King Abdullah University of Science and Technology (KAUST), Computational Bioscience Research Center (CBRC), Thuwal 23955-6900, Saudi Arabia.

^2^King Abdullah University of Science and Technology (KAUST), Infocloud Group, Computer, Electrical and Mathematical Sciences and Engineering Division (CEMSE), Thuwal 23955-6900, Saudi Arabia.

Author Emails:

Othman Soufan: othman.soufan@kaust.edu.sa,

Wail Ba-alawi: wail.baalawi@kaust.edu.sa,

Moataz Afeef: moataz.afeef@kaust.edu.sa,

Magbubah Essack: magbubah.essack@kaust.edu.sa,

Panos Kalnis: panos.kalnis@kaust.edu.sa,

Vladimir B. Bajic: vladimir.bajic@kaust.edu.sa

* Corresponding author: Vladimir B. Bajic: vladimir.bajic@kaust.edu.sa

**Additional File 2**

In the following discussion, we show results using different validation methods and extensive comparison of several methods. From the class of binary relevance solutions, we use BR-RF method in this section of extended comparisons since it has achieved higher results than BR-SVM and BR-KNN as shown in the main manuscript.

Here, we describe results of our experimental studies over five large HTS assays composed of more than 1.4 million interactions and more than 400,000 chemical compounds from the PubChem BioAssay Database (1). In Table 1, we list results using holdout validation method. A full range of holdout parts of data is used to challenge and test the models. Ranging from 80% to only 20% of original data used to train the models, DRABAL achieved the highest results in all cases. On average, DRABAL improved F_1_Score by 6.8% and 22.24% when compared to BR-RF and CC-MLE, respectively. BR-RF is a very hard to beat baseline especially when the number of target labels is considerably small (2). When 50% to 80% of data is used for testing a model, results over such portions of samples can give a strong indication about generalization ability of the model. Relative to first holdout experiment (i.e. 80/20), performance dropped by 52.12% using DRABAL when considering the last holdout experiment (i.e. 20/80). Such decline in performance is expected with much smaller number of samples used for training. However, BR-RF reflects more sensitivity to such changes with a relative decline in performance of 72.5%.

Table 1: Comparison of methods across five different datasets using holdout validation. First number in parenthesis corresponding to portion of training while the second number highlight the portion used for testing. For example, (20/80) indicates that 20% of original data was used for training and 80% for testing.

| **Method** | **F_1_Score (80/20)** | **F_1_Score (70/30)** | **F_1_Score (60/40)** | **F_1_Score (50/50)** | **F_1_Score (40/60)** | **F_1_Score (30/70)** | **F_1_Score (20/80)** |
| --- | --- | --- | --- | --- | --- | --- | --- |
| **DRABAL** | **51.83%** | **49.51%** | **47.02%** | **44.88%** | **42.03%** | **39.18%** | **34.07%** |
| **BR-RF** | 45.38% | 43.75% | 40.44% | 38.26% | 35.40% | 31.47% | 26.30% |
| **CC-MLE** | 28.85% | 27.08% | 24.51% | 22.14% | 20.59% | 16.68% | 13.01% |

An ideal scenario to evaluate models and test generalization ability of the models is to extract real data from another external repository of chemical-target interactions. HTS assays hold information including specific details about experimental protocols and phenotypes of interaction. We could not find external records that match such conditions and hold novel interaction that were not reported earlier. Therefore, in order to simulate a scenario of testing the models over external novel cases, we generated synthetic samples at several distance ranges from the portion of the original data. The construction of synthetic samples is challenging for few reasons. The data we have is a multi-label classification type of data and therefore, we do not have explicitly positive and negative classes that we can exploit for generating new samples. In the multi-label scenario, the same compound can be reported as active in one HTS assay and as inactive in another assay. A decision about to which group this compound belongs to in the space is, in turn, not direct. This should be taken into account when generating synthetic samples. Also, defining synthetic samples would need certain assumptions like which distance measure to use.

We randomly select 30% of the original data (this makes a set S30), and for each sample in S30 we generate a synthetic sample at a distance 1, 2, …, 20 from the selected S30 sample. In order to do this, we reverse the Euclidean distance formula to get feature values of a new sample that satisfy such given distances (see Appendix for mathematical details). The target labels we assign for each synthetic sample are the target labels of the corresponding sample in the original data. We end up with 20 synthetic datasets. Each of these datasets is then used to test all methods. To make the synthetic data independent from the training data, we excluded S30 set from the original data and trained models on this reduced dataset. Results are shown Figure S1.

Figure S1 shows that in all cases DRABAL achieved higher performance scores than BR-RF and CC-MLE (i.e. the second best and the third best methods in our study, respectively). We take BR-RF as a representative for other BR class of solutions since it achieved the highest results among them (see reported results in the main manuscript).


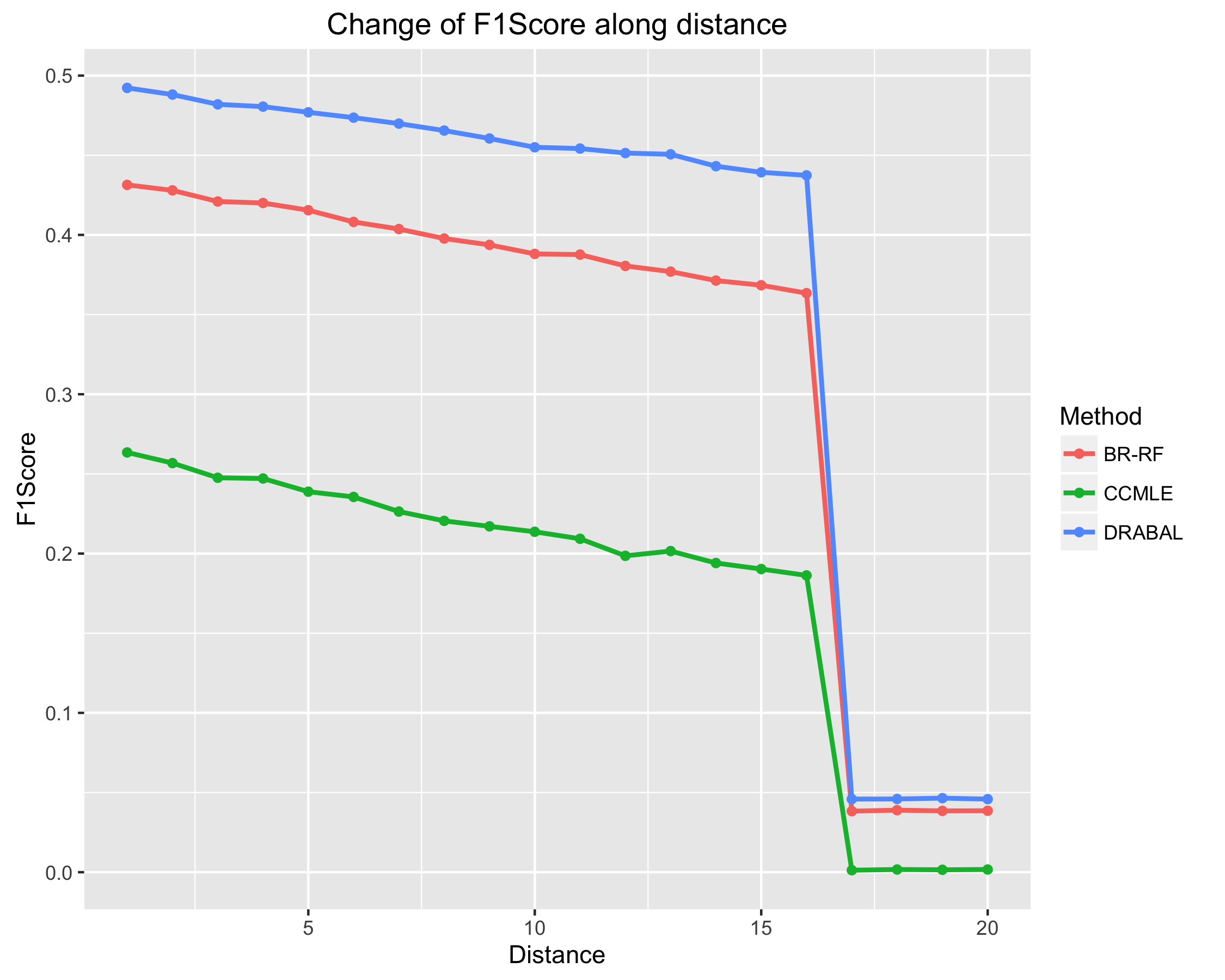


Figure S1: F_1_Score over distance comparison of methods. Synthetic data is generated at fixed distances from original data to serve as external source of evaluation. A portion of 30% of the original data is hidden from training and used to generate a more challenging set of synthetic samples for testing.

Another set of experiments we performed to assess capabilities of the models is based on evaluating performance at a distance from the centroid of all data. We compute centroid of all data, and then find a distance threshold such that 50% of data will be within this distance. This part of the data will be considered as the training set. After that, we take 20 groups of 1000 sampled data points where each group resides at a further distance from the training set. This helps in evaluating performance over distance using a dissimilarity radius from known training samples. Figure S2 shows the plot of F_1_Score over 20 distant testing groups. Group 1 is the closest to the training part while group 20 is the furthest. As the figure illustrates, our method DRABAL achieved the highest performance at all distance groups. Overall, the performance degrades as distance increases and this is normal since testing samples are chosen to be far in the space from the training cases, imposing a more difficult classification task at larger distances. The oscillation in the graph is suspected to result from choosing some points as testing samples that are distant from centroid but not necessarily from all training samples.


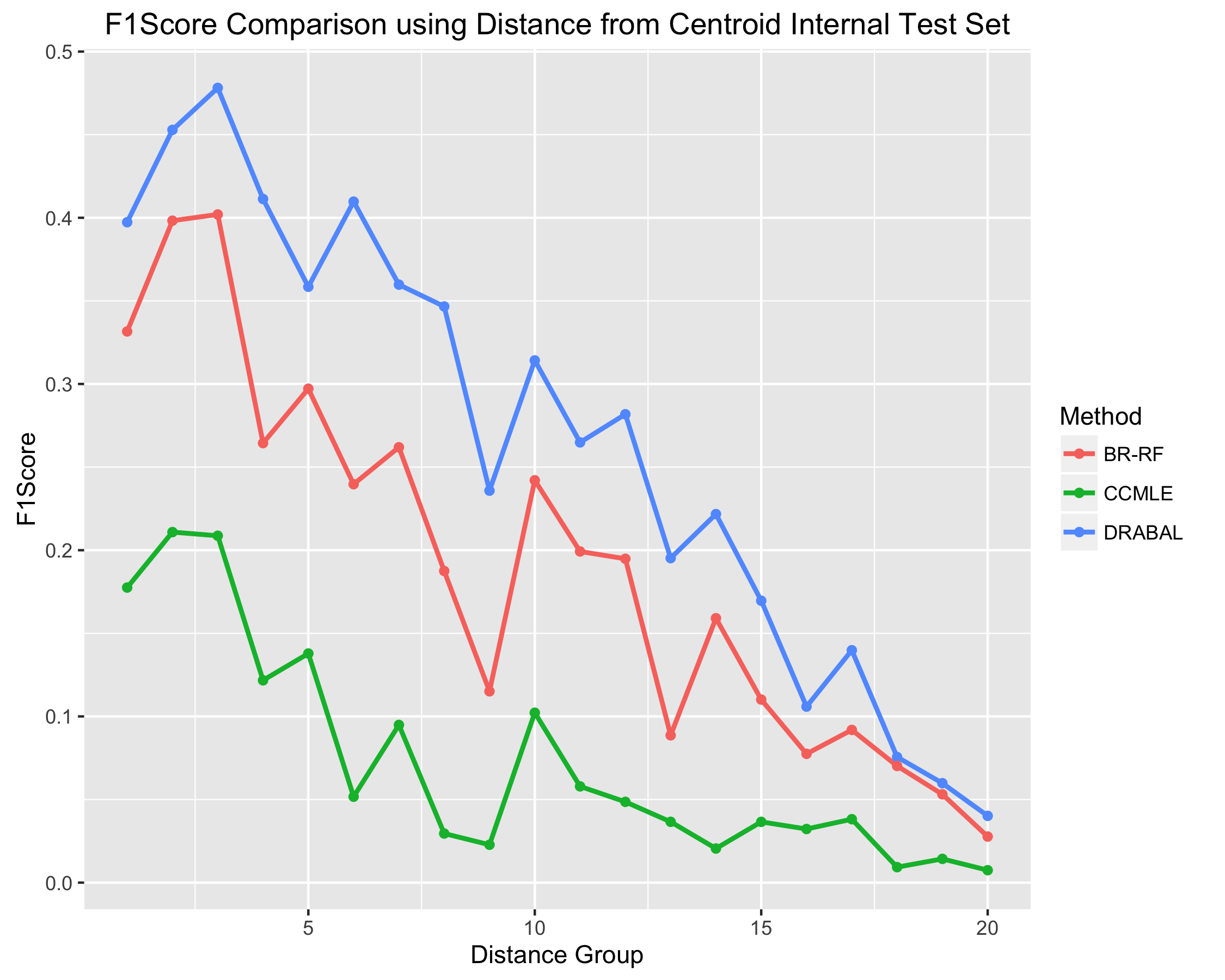


Figure S2: F_1_Score over distance comparison of methods. Data is partitioned into several groups based on distance from the centroid of all samples.

# Appendix

Given point $r=(r_{1},r_{2},\ldots,r_{n})$ which is taken from the original data, we want to compute point $s=(s_{1},s_{2},\ldots,s_{n})$ which is at a predefined distance from $r$. The components of both points (i.e. $r_{i}$ or $s_{i}$) represent feature values of each point. The following equation illustrates how we derive computation to generate a novel synthetic sample $s$. Note that we make use of an assumption that reflects a distance taken at hypercube’s boundaries from the training samples $r$. Avoiding such assumption would then force us to solve a difficult optimization problem to find points at exact distance from another point or even at a minimum distance threshold. Such optimization is intractable for the large number of samples we have in our dataset.

$$distance\left( r,s \right)=d\left( r,s \right)=\sqrt{\sum_{i=1}^{n} ({r_{i}-s_{i})}^{2}}$$

${d\left( r,s \right)}^{2}=\sum_{i=1}^{n} ({r_{i}-s_{i})}^{2}$(squaring both sides)

Let’s now assume that $r_{1}-s_{1}=r_{2}-s_{2}=\ldots=r_{n}-s_{n}=a$. We make this assumption to simplify derivation of a new samples based on distance as an input. Also, if we do not put this assumption, we end up having $n$ number of unknowns which is intractable to solve. This assumption reflects defining a synthetic sample residing at some boundary of a hypercube from the training sample:

${d\left( r,s \right)}^{2}=\sum_{i=1}^{n} ({a)}^{2}$(using the assumption)

${d\left( r,s \right)}^{2}=na^{2}$ (definition of summation)

$\frac{{d\left( r,s \right)}^{2}}{n}=a^{2}$ (divide by $n$)

$\sqrt{\frac{{d\left( r,s \right)}^{2}}{n}}=a$ (take square root for both sides)

$$\frac{d\left( r,s \right)}{\sqrt{n}}=a$$

Now, given any distance $d\left( r,s \right)$, we can compute $a$, which is the value we should subtract or add for each feature value to preserve any desired distance $d\left( r,s \right)$. Based on this derivation, we were able to define 20 different testing sets setting exactly at specific distances from the training data.

# References

1. Wang Y, Xiao J, Suzek TO, Zhang J, Wang J, Bryant SH. PubChem: a public information system for analyzing bioactivities of small molecules. Nucleic acids research. 2009:gkp456.

2. Read J, Pfahringer B, Holmes G, Frank E. Classifier chains for multi-label classification. Machine learning. 2011;85(3):333-59.
