# Supplementary material for: DRABAL: novel method to mine large high-throughput screening assays using Bayesian active learning
Source: J Cheminform. 2016 Nov 10;8:64. doi: 10.1186/s13321-016-0177-8 (PMC5105261; doi:10.1186/s13321-016-0177-8)
Supplement: Supplementary file 4 — Additional file 4.Pseudocode of DRABAL. [file 13321_2016_177_MOESM4_ESM.pdf]

Algorithm 1: DRABAL for Multi-label Classification

```
Input: Data matrix D, Labels matrix L, Base Classifier C
Output: BNsorted
/* Output: Nodes of BNsorted graph holds trained models for
   each label in L                                              */
ActualScores = [ ][ ] ;      // Initialize an empty matrix to hold
    predicted probability scores
BNgraph = learnBayesianNetworkStructure(L);  // Any algorithm for
    learning a proper DAG structure characterizing distribution
    of L can be used.
BNsorted = topologicalSort( BNgraph );
for each node x ∈ BNsorted do
    TrainData = D;           // each step re-initialize TrainData
    parentlist = getParents(x);
    if length(parentlist) ≠ 0 then
        for each parent p ∈ parentlist do
            /* Add predicted probability scores stored in parent
               nodes to the original data matrix as an extra
               feature column                                   */
            TrainData = joinData(TrainData, ActualScores[:][p.idx])
        end
    end
    clf = C.fit(TrainData, L); // clf can be any trained classifier
    ActualScores[:][x.idx] = clf.computeProbs(TrainData) ;  // x.idx is
        the corresponding index of node x in matrix L
    x.clf = clf ;            // store the trained model for node x
end
```
